# Fermentation of Structurally Defined Alginate Oligosaccharides by the Human Gut Microbiota Enriched in *Bifidobacterium*, *Bacteroides*, *Faecalibacterium*, or *Blautia*

**DOI:** 10.3390/md24070239

**Published:** 2026-07-07

**Authors:** Siyu Liu, Yuchen Wu, Youjing Lv, Meng Shao, Depeng Lv, Quancai Li, Qingsen Shang

**Affiliations:** 1 Key Laboratory of Marine Drugs of Ministry of Education, Shandong Key Laboratory of Glycoscience and Glycotherapeutics, School of Medicine and Pharmacy, Ocean University of China, Qingdao 266003, China; 13697890217@163.com (S.L.); wyc7600@stu.ouc.edu.cn (Y.W.); lvyoujing1988@163.com (Y.L.); 2 Laboratory for Marine Drugs and Bioproducts, Qingdao Marine Science and Technology Center, Qingdao 266237, China; 3 Marine Biomedical Research Institute of Qingdao, Qingdao 266071, China; shaomeng@ouc.edu.cn (M.S.); lvdepeng@ouc.edu.cn (D.L.)

**Keywords:** alginate oligosaccharides, prebiotic, gut microbiota, enterotype, short-chain fatty acids, fermentation, personalized nutrition

## Abstract

Alginate oligosaccharides (AOS) are attractive candidates for prebiotic development, yet how oligosaccharide structure and baseline microbial community composition interact to shape fermentation remains an open question. In this study, we stratified fecal microbiota from healthy donors into operational genus-predominance groups (*Bifidobacterium*, *Bacteroides*, *Faecalibacterium*, or *Blautia*) and selected representative samples for in vitro fermentation of six structurally distinct AOS preparations (SAOS-1, SAOS-2, OAOS, UAOS, SMOS, and SGOS). Substrate consumption, short-chain fatty acid (SCFA) production, and shifts in microbial community structure were profiled. The six preparations differed in structural type, number-average molecular weight, and average degree of polymerization. Among them, SAOS-1 exhibited the most consistent utilization across all four groups and yielded the highest total SCFA production. SAOS-1 fermentation also attenuated inter-group community divergence and enriched several beneficial or functionally relevant taxa, including *Bacteroides* and *Faecalibacterium*. Interestingly, the magnitude and direction of microbial responses remained enterotype-dependent, with the *Bacteroides*-predominant group assembling the most complex fermentative consortium. These findings demonstrate that AOS structure and baseline microbial ecology jointly dictate fermentation outcomes, positioning SAOS-1 as a strong candidate for precision prebiotic development. This structure–community interaction paradigm provides a rational basis for the targeted deployment of marine oligosaccharides in personalized gut health strategies.

## 1. Introduction

Alginate oligosaccharides (AOS) are structurally heterogeneous products derived from the depolymerization of alginate, a brown-algal polysaccharide composed of β-D-mannuronic acid (M) and α-L-guluronic acid (G) arranged as M-, G-, and MG-blocks [1,2]. Their structural features are determined by both the parent alginate and the preparation process, including depolymerization and downstream fractionation. As a result, AOS preparations may differ in molecular-weight distribution, degree of polymerization (DP), saturation state, terminal chemistry, oxidation state, and M/G composition [3,4]. These differences are important because they may affect microbial recognition, enzymatic cleavage, transport, substrate utilization, and fermentation outputs such as short-chain fatty acid (SCFA) production and microbial community shifts [5]. Therefore, structurally distinct AOS preparations should not be regarded as interchangeable substrates for gut microbiota fermentation.

The six AOS preparations used in this study represent different structural categories. Saturated alginate oligosaccharides (SAOS-1, SAOS-2) were both prepared from sodium alginate by enzymatic hydrolysis followed by membrane fractionation, including ultrafiltration to remove high-molecular-weight residues and nanofiltration to separate different molecular-weight fractions. Thus, SAOS-1 and SAOS-2 share the same saturated alginate-derived origin but differ mainly in chain length and molecular-weight distribution: SAOS-1 is a relatively higher-molecular-weight fraction of approximately 800–1000 Da with a DP range mainly of 2–10, whereas SAOS-2 is a lower-molecular-weight fraction of approximately 400 Da with a DP range mainly of 2–3 or 2–4. Unsaturated alginate oligosaccharides (UAOS) are characterized by a C4–C5 unsaturated uronate residue generated during alginate lyase-mediated β-elimination [6], whereas oxidized alginate oligosaccharides (OAOS) are produced by oxidative degradation and contain oxidative structural modifications [7]. Saturated mannuronate oligosaccharides (SMOS) and Saturated guluronate oligosaccharides (SGOS) are saturated mannuronate-rich and guluronate-rich oligosaccharide fractions, respectively [8]. Together, these six substrates provide a useful model for evaluating how AOS chain length, saturation state, oxidation state, terminal chemistry, and M/G composition influence fermentation by different baseline gut microbiota [9].

From a nutritional perspective, AOSs are attractive candidate prebiotics. The current International Scientific Association for Probiotics and Prebiotics (ISAPP) definition emphasizes that a prebiotic should be selectively utilized by host microorganisms and confer a health benefit [10]. For dietary fibers such as AOS, the most relevant microbial outputs include short-chain fatty acids (SCFAs), especially acetate, propionate, and butyrate. These metabolites support epithelial energy metabolism, gut barrier function, immune regulation, and systemic metabolic homeostasis [11,12,13,14]. However, selectivity is central to the prebiotic concept: a substrate should not merely be fermentable, but should be preferentially used by specific microbial groups or consortia that generate beneficial ecological or metabolic consequences. Alginate-based materials are also closely associated with probiotic protection during gastrointestinal transit. Because gastric acidity and bile exposure can reduce probiotic viability, alginate beads and alginate-based hydrogel microcapsules have been widely used to encapsulate probiotic bacteria and improve their survival under simulated gastric and intestinal conditions. Previous studies showed that alginate coating or alginate-based microencapsulation enhanced the survival of probiotic strains such as *Lactobacillus acidophilus* and *Lactobacillus rhamnosus* during simulated gastrointestinal exposure [15,16]. These studies provide useful background for alginate–bacteria interactions in the gastrointestinal environment, but the selective fermentation of structurally distinct AOS by different baseline gut microbiota remains unclear.

A growing body of evidence indicates that alginate degradation in the human gut is mediated by a limited set of specialist microorganisms. Several *Bacteroides* species encode alginate utilization loci containing polysaccharide lyases, transporters, and regulatory components that allow them to capture and degrade alginate-derived glycans [17,18]. In vitro colon-simulation and strain-based studies further suggest that AOS can modulate complex gut microbial communities and that species such as *Bacteroides xylanisolvens* may act as keystone degraders for alginate and AOS fermentation [19,20]. These primary degraders may release oligosaccharides or fermentation intermediates that are subsequently consumed by secondary fermenters, including butyrate-producing *Firmicutes*, thereby linking alginate foraging with community-level cross-feeding [21,22].

Nevertheless, human responses to dietary fibers and prebiotics are highly individualized. The original enterotype framework classified human gut microbiota into community configurations enriched in *Bacteroides*, *Prevotella*, or *Ruminococcus*, and later work linked *Bacteroides*- and *Prevotella*-enriched communities with long-term dietary patterns [23,24,25]. Although enterotypes should not be interpreted as rigid biological categories, they remain useful as a simplified ecological descriptor of baseline microbiota structure and potential carbohydrate-utilization capacity. Previous studies from our group and others showed that *Bacteroides*-dominated microbiota generally ferment alginate, polymannuronate, polyguluronate, and several marine oligosaccharides more efficiently than *Prevotella*- or *Escherichia*-dominated microbiota [26,27]. These observations support the concept that baseline microbial ecology may determine whether a given marine oligosaccharide functions as a broad-spectrum or personalized prebiotic.

In the present study, we used a function-oriented, genus-predominance strategy rather than a canonical enterotype classifier. Healthy volunteers were first screened by 16S rRNA gene sequencing, and fecal microbiota were grouped according to the dominant genus among four health-relevant and carbohydrate-metabolizing genera: *Bifidobacterium*, *Bacteroides*, *Faecalibacterium*, and *Blautia.* This approach does not claim to redefine classical enterotypes; instead, it provides an operational framework to test whether different baseline microbial configurations show distinct preferences for structurally defined AOS. We compared the in vitro fermentation of six AOS preparations, namely SAOS-1, SAOS-2, OAOS, SMOS, and SGOS, by representative microbiota from each group, quantified substrate utilization and SCFA production, and further examined the community-modulating effects of the most preferred substrate, SAOS-1. We hypothesized that AOS structure and baseline microbial configuration jointly determine fermentation efficiency, metabolite profiles, and selective enrichment of beneficial bacterial taxa.

## 2. Results

### 2.1. Four Functional Enterotypes Identified Based on Genus Predominance

A total of 32 fecal samples were classified into four enterotypes according to the most abundant genus among *Bifidobacterium*, *Bacteroides*, *Faecalibacterium*, and *Blautia*. The genus-level composition of each enterotype is shown in Figure S1A. Principal coordinate analysis (PCoA) based on Bray–Curtis distances revealed a clear separation among the four groups, indicating distinct microbial community structures (Figure S1B).

Among the 32 volunteers, 8, 9, 7, and 8 individuals were classified as *Bifidobacterium*, *Bacteroides*, *Faecalibacterium*, and *Blautia* enterotypes, respectively. From each group, the three subjects with the highest relative abundance of the corresponding dominant genus were selected for fermentation. For subsequent in vitro fermentation experiments, three representative samples were selected from each enterotype, giving a total of 12 samples. The selected samples were: *Bifidobacterium*: FZ19, FZ7, FZ17; *Bacteroides*: FZ10, FZ18, FZ16; *Faecalibacterium*: FZ26, FZ23, FZ14; *Blautia*: FZ4, FZ31, FZ20. These samples were used to investigate the fermentation of six structurally defined AOS.

It should be noted that the term “enterotype” in this study was used as an operational descriptor of genus-predominance-based microbial configurations rather than as a canonical enterotype classification. This strategy was adopted to stratify fecal microbiota according to dominant, functionally relevant genera and to evaluate whether baseline microbial composition influenced AOS fermentation capacity.

### 2.2. Enterotype-Specific Consumption of Six AOS

The six AOS preparations used in this study differed in structural type, number-average molecular weight (Mn), and average degree of polymerization (DP) (Table 1 and Figure S2). SAOS-1 and SAOS-2 represented saturated alginate oligosaccharide fractions with different molecular sizes, whereas UAOS, OAOS, SMOS, and SGOS represented unsaturated, oxidized, saturated mannuronate, and saturated guluronate oligosaccharides, respectively. These structural differences provided the basis for comparing enterotype-dependent fermentation responses.

**Figure 1:**
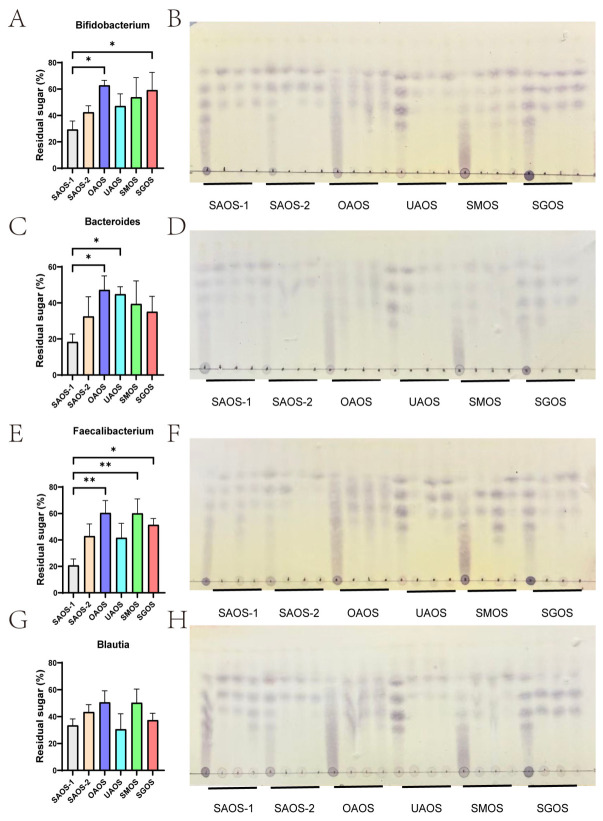
Fermentation and utilization of six AOSs (SAOS-1, SAOS-2, OAOS, UAOS, SMOS, and SGOS) by four different human gut enterotypes. Each fermentation group contained three fecal samples. Utilization of the oligosaccharides during fermentation (**A**,**C**,**E**,**G**). TLC analysis of oligosaccharides before and after fermentation by different enterotypes (**B**,**D**,**F**,**H**). For each group, the first lane (Con) represents the control medium at the start of fermentation. All fermentation experiments were performed in triplicate. Data are expressed as mean ± SEM. Statistical analysis was performed using ANOVA with post hoc Tukey’s multiple comparisons test (* *p* < 0.05, and ** *p* < 0.01).

After 48 h of fermentation, the consumption of the six AOS was assessed by total carbohydrate measurement (Figure 1A,C,E,G). SAOS-1 was the most efficiently utilized substrate across all four enterotypes, leaving the lowest residual sugar in every case. By contrast, SAOS-2 and OAOS were generally less fermented, particularly in the *Bifidobacterium* and *Faecalibacterium* groups. The capacity to degrade the homopolymeric SMOS and SGOS varied markedly with enterotype. The *Bacteroides*-enriched microbiota exhibited the broadest activity, efficiently utilizing both SMOS and SGOS. The *Blautia* group also showed moderate degradation of these substrates, whereas the *Bifidobacterium* and *Faecalibacterium* groups were less effective.

Thin-layer chromatography (TLC) analysis visually confirmed the consumption patterns (Figure 1B,D,F,H). In all enterotypes, the SAOS-1 band nearly disappeared after fermentation. Notably, the bands for SMOS and SGOS were substantially weakened only in the *Bacteroides* group, consistent with its superior ability to degrade these homopolymeric oligosaccharides.

### 2.3. SCFA Production

**Figure 2:**
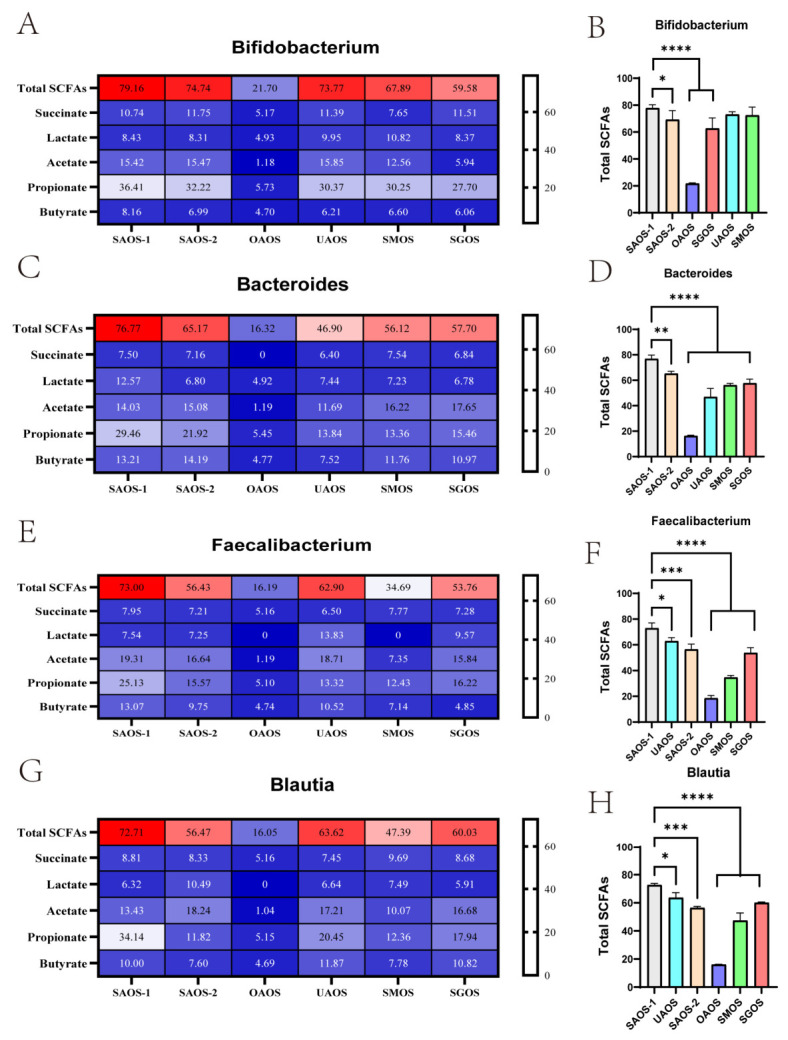
Production of SCFAs by four different human gut enterotypes during fermentation of six AOS. (**A**,**B**) Heatmap of SCFAs and bar graph of total SCFAs for the *Bifidobacterium* enterotype. (**C**,**D**) Heatmap of SCFAs and bar graph of total SCFAs for the *Bacteroides* enterotype. (**E**,**F**) Heatmap of SCFAs and bar graph of total SCFAs for the *Faecalibacterium* enterotype. (**G**,**H**) Heatmap of SCFAs and bar graph of total SCFAs for the *Blautia* enterotype. Each fermentation group contained three fecal samples. The numerical values shown in the heatmap indicate SCFA concentrations, while the color gradient represents the relative magnitude of SCFA production. All fermentation experiments were performed in triplicate. Data are expressed as mean ± SEM. Statistical analysis was performed using ANOVA with post hoc Tukey’s multiple comparisons test. (* *p* < 0.05, ** *p* < 0.01, *** *p* < 0.001, and **** *p* < 0.0001). We have revised the legend of Figure 2 to clarify the meaning of the numerical values shown in the heatmap, as well as the corresponding units and color scale.

Consistent with the carbohydrate utilization patterns, SAOS-1 yielded the highest total SCFAs among the six oligosaccharides in all four enterotypes, while OAOS consistently produced the lowest levels (Figure 2B,D,F,H). This agreement between substrate consumption and SCFA production further confirms that SAOS-1 was the most efficiently fermented substrate regardless of the dominant genus.

The SCFA profiles differed markedly among enterotypes. In the *Bacteroides* group, fermentation of SAOS-1 and SGOS generated a higher proportion of butyrate compared with the other three enterotypes (Figure 2C,D). In contrast, the *Bifidobacterium* and *Faecalibacterium* microbiotas were characterized by elevated propionate levels, whereas the *Blautia* group showed an intermediate pattern (Figure 2A,B,E–H). These differences suggest that the fermentation characteristics and prebiotic potential of the six AOS are enterotype-dependent. Notably, the ability to produce butyrate, a key metabolite for gut health, was most pronounced in the *Bacteroides* microbiota when supplied with SAOS-1 or SGOS. Given that SAOS-1 consistently showed the highest fermentation efficiency and SCFA production across all enterotypes, it was selected as the representative substrate for further analysis of gut microbiota modulation.

### 2.4. SAOS-1 Fermentation Induces Enterotype-Specific Diversity Changes with Convergent Community Structure

**Figure 3:**
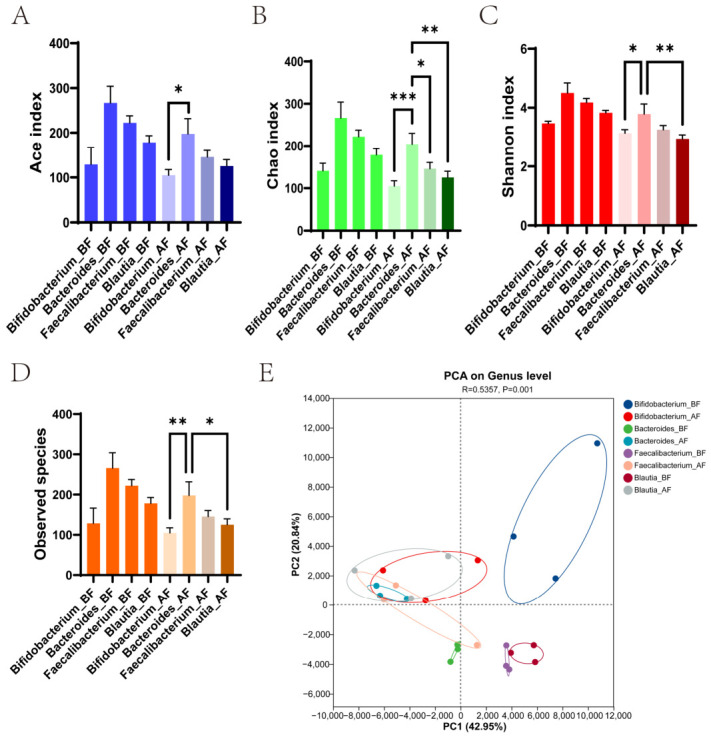
Diversity analysis of gut microbiota before and after fermentation of SAOS-1 by four different human gut enterotypes. BF, before fermentation; AF, after fermentation with SAOS-1. α-Diversity analysis: (**A**) Ace index, (**B**) Chao1 index, (**C**) Shannon index, and (**D**) Observed species. (**E**) PCA score plot of gut microbiota before and after SAOS-1 fermentation across different enterotypes. (* *p* < 0.05, ** *p* < 0.01, and *** *p* < 0.001).

To investigate how SAOS-1 reshapes the gut microbial community across different enterotypes, α- and β-diversity were compared before and after fermentation (Figure 3A–E). After 48 h of fermentation with SAOS-1, α-diversity indices (Ace, Chao1, Shannon, and observed species) were compared among the four enterotypes (Figure 3A–D). The *Bacteroides* group exhibited significantly higher values for all four indices than the *Bifidobacterium*, *Faecalibacterium*, and *Blautia* groups. The other three enterotypes showed comparably lower diversity levels, with no significant differences among them. This indicates that the gut microbiota of the *Bacteroides* enterotype maintained greater species richness and evenness after SAOS-1 fermentation, suggesting a distinct ecological response to this oligosaccharide.

β-Diversity analysis by principal component analysis (PCA) revealed a different pattern (Figure 3E). While the four pre-fermentation (BF) groups formed clearly separated clusters according to their original enterotype, the post-fermentation (AF) samples shifted toward each other, resulting in substantially reduced inter-enterotype distances. This convergence implies that SAOS-1 fermentation drove the microbial communities of different groups toward a more similar overall structure, despite persistent alpha-diversity differences. Collectively, SAOS-1 showed broad fermentability and induced partial convergence of microbial community structure across different genus-predominance groups, while the *Bacteroides* group retained higher microbial diversity after fermentation.

### 2.5. SAOS-1 Fermentation Reshaped Gut Microbiota Composition with a Distinct Response in the Bacteroides Enterotype

**Figure 4:**
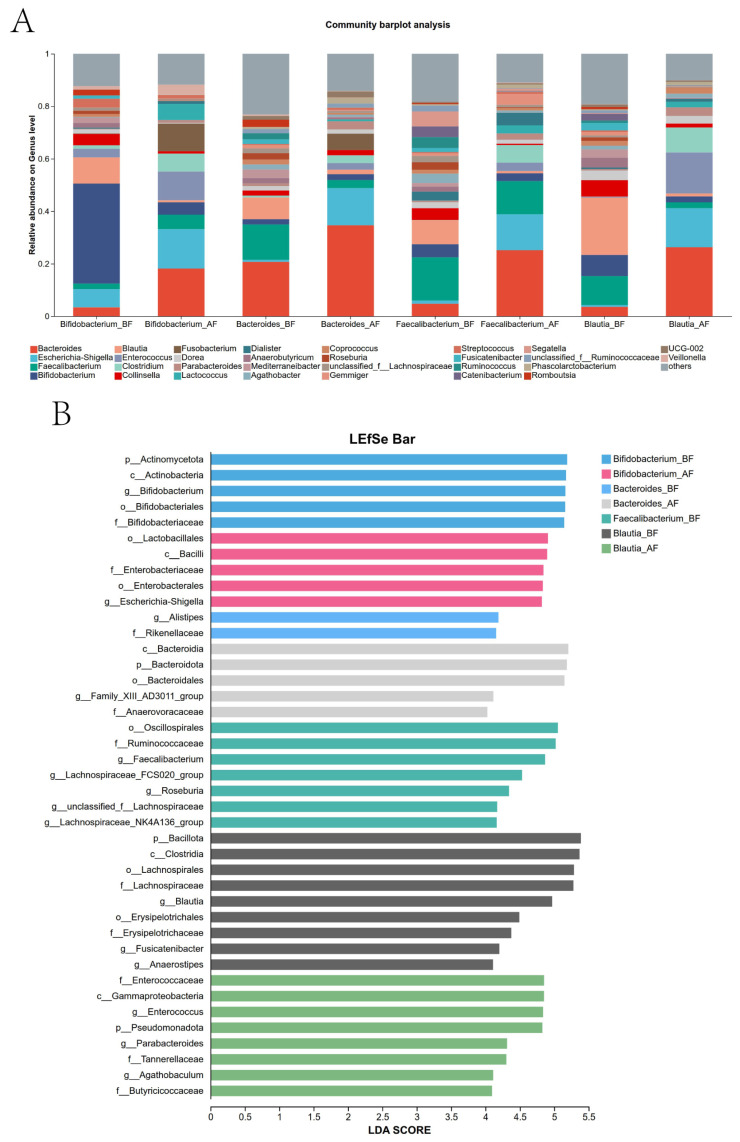
Composition analysis of gut microbiota before and after fermentation of SAOS-1 by four different human gut enterotypes. BF, before fermentation; AF, after fermentation with SAOS-1. (**A**) Bar plot of microbial composition at the genus level. (**B**) LDA of the microbiota during SAOS-1 fermentation by LEfSe. The analysis was performed at the genus level. Only taxa with an LDA score > 2 are listed.

Genus-level composition and LEfSe analysis were compared among the eight groups (Figure 4A,B). SAOS-1 fermentation reduced the relative abundance of the dominant genus in each enterotype, including *Bifidobacterium* spp. in the *Bifidobacterium* group, *Faecalibacterium* spp. in the *Faecalibacterium* group, and *Blautia* spp. in the *Blautia* group. Concurrently, *Bacteroides* spp. and *Faecalibacterium* spp. were enriched across all four groups after fermentation (Figure 4A). This uniform enrichment supports the β-diversity convergence described in Figure 3E.

The *Bacteroides* group showed a different pattern. *Bacteroides* spp. remained the most abundant genus after fermentation, but its proportion decreased. Several butyrate-producing genera, including *Faecalibacterium* spp., *Roseburia* spp., and members of the family *Lachnospiraceae*, including *Blautia* spp., *Dorea* spp., and *Coprococcus* spp., expanded notably (Figure 4A). LEfSe analysis further identified that *Bacteroides*_AF was characterized by the co-occurrence of *Bacteroides* spp., *Faecalibacterium* spp., and *Lachnospiraceae* spp. This discriminant signature was absent in the other three AF groups (Figure 4B). In contrast, the *Bifidobacterium*, *Faecalibacterium*, and *Blautia* groups did not develop such a diverse consortium of butyrate producers after fermentation.

Thus, the *Bacteroides* enterotype exhibited a unique ecological response. Its native *Bacteroides* spp. population did not simply dominate but instead supported a more complex network of cross-feeding butyrate-producing bacteria. This property likely explains the significantly higher α-diversity retained by the *Bacteroides* group after fermentation. Therefore, SAOS-1 did not simply induce community convergence but also appeared to favor a more complex consortium of SCFAs-associated taxa in the *Bacteroides* group.

### 2.6. SAOS-1 Induces Enterotype-Specific Enrichment of Signature Bacterial Taxa

**Figure 5:**
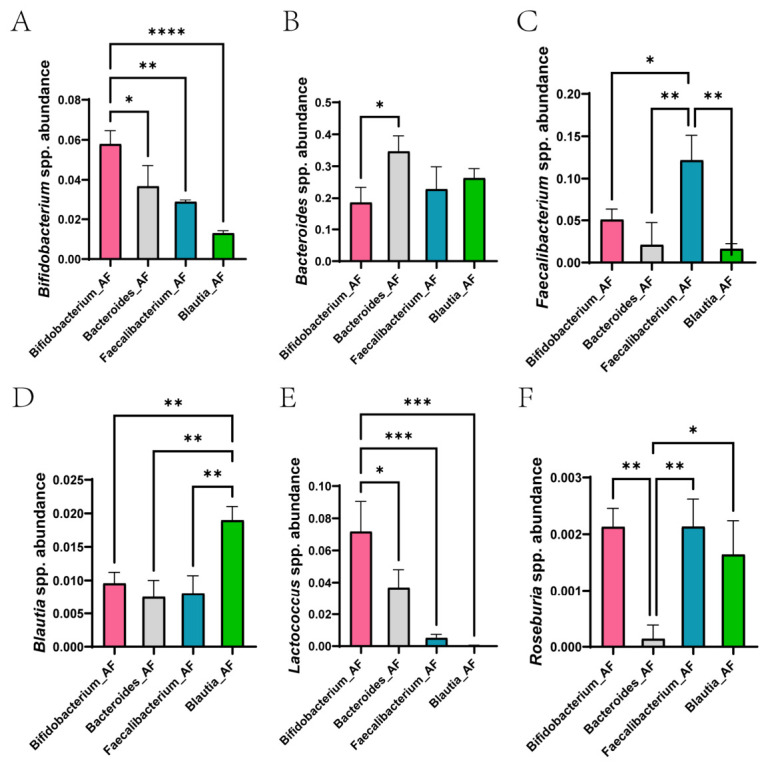
Enterotype-dependent effects of SAOS-1 fermentation on selected bacterial genera. BF, before fermentation; AF, after fermentation with SAOS-1. Relative abundances of (**A**) *Bifidobacterium* spp., (**B**) *Bacteroides* spp., (**C**) *Faecalibacterium* spp., (**D**) *Blautia* spp., (**E**) *Lactococcus* spp., and (**F**) *Roseburia* spp. (* *p* < 0.05, ** *p* < 0.01, *** *p* < 0.001, and **** *p* < 0.0001).

To determine whether SAOS-1-induced changes in selected health-associated taxa depended on the initial microbial configuration, the relative abundances of six representative genera were compared across the four post-fermentation groups (Figure 5A–F). SAOS-1 preferentially promoted the growth of the dominant genus originally present in each enterotype. In the *Bifidobacterium* group, *Bifidobacterium* spp. and *Lactococcus* spp. reached their highest levels among all groups. In the *Bacteroides* group, *Bacteroides* spp. itself remained the most abundant. In the *Faecalibacterium* group, *Faecalibacterium* spp. was strongly enriched. In the *Blautia* group, *Blautia* spp. showed the highest abundance. In contrast, *Roseburia* spp. was enriched in the *Bifidobacterium* and *Faecalibacterium* groups but was nearly absent in the *Bacteroides* and *Blautia* groups.

These results indicate that the microbiota-modulating effect of SAOS-1 is highly selective. Each genus-predominance group tended to retain or enrich its own signature taxon, rather than showing a uniform increase in all selected health-associated genera. The *Bacteroides* enterotype, despite its ability to foster a diverse butyrate-producing consortium, did not enrich *Roseburia* spp. This enterotype-dependent selectivity underscores that the response to a prebiotic is shaped by the resident gut microbiota, supporting the concept of personalized prebiotic interventions.

## 3. Discussion

This study demonstrates that structurally defined AOS are not equivalent substrates for human gut microbiota and that their fermentation profiles are strongly influenced by the baseline microbial configuration. Among the six tested AOS, SAOS-1 showed the most consistent and extensive utilization across all four functional enterotype groups and produced the highest total SCFA levels. This finding is important because previous work has mainly compared alginate, PM, PG, or broader classes of marine oligosaccharides across conventional enterotypes [24,25], whereas the present study directly links fine structural variation in AOS with enterotype-dependent fermentation performance.

The superior fermentability of SAOS-1 may be related to a favorable combination of saturated structure and chain-length distribution. Compared with chemically modified or unsaturated AOS, SAOS-1 may retain greater compatibility with microbial alginate-degrading systems. Oxidation can introduce ring opening and terminal chemical modifications, which may reduce recognition by transporters or lyases, whereas unsaturated terminal residues generated by lyase treatment may require specialized downstream enzymes for complete assimilation [17,18]. The relatively poor fermentation of OAOS in this study is consistent with the idea that excessive chemical modification can decrease microbial accessibility even when solubility is improved. However, SAOS-1 and SAOS-2 were both saturated preparations but differed in fermentation performance, indicating that saturation alone is insufficient to explain substrate preference. As detailed in the Methods (Section 4.2), the six AOS preparations differ not only in saturation but also in molecular weight, DP, M/G ratio, and terminal chemistry, which may collectively contribute to the preferential fermentation of SAOS-1.

A second major finding is that SAOS-1 produced both convergent and divergent ecological effects. After SAOS-1 fermentation, β-diversity distances among the four groups decreased, and *Bacteroides* and *Faecalibacterium* tended to be enriched across groups. This convergence suggests that SAOS-1 selects for a limited set of microbial functions related to alginate-derived oligosaccharide utilization and SCFA production. At the same time, the Bacteroides group retained the highest α-diversity and showed a more complex consortium involving *Bacteroides* spp., *Faecalibacterium* spp., and *Lachnospiraceae* spp.-related taxa. This pattern is biologically plausible because *Bacteroides* spp. species often act as primary degraders of complex glycans through polysaccharide-utilization loci, while butyrate-producing *Firmicutes* can benefit from acetate, succinate, lactate, or released oligosaccharides generated during primary degradation [21,22]. Thus, SAOS-1 may act not only as a direct substrate for alginate-degrading *Bacteroides* spp. but also as an ecological trigger for cross-feeding networks that support butyrate production.

The selective enrichment of health-associated and functionally relevant genera further supports an enterotype-dependent interpretation of SAOS-1 prebiotic potential. In the *Bifidobacterium* group, *Bifidobacterium* spp. and *Lactococcus* spp. reached relatively high levels; in the *Faecalibacterium* and *Blautia* groups, the corresponding baseline signature genera were preferentially maintained or enriched; and in the *Bacteroides* group, *Bacteroides* spp. remained a central member while additional butyrate-associated taxa expanded. These results agree with the broader literature showing that baseline microbiota composition, habitual fiber intake, and the presence of carbohydrate-active guilds can predict individual responses to prebiotic fibers [28,29]. Therefore, the same oligosaccharide may have different ecological endpoints depending on whether the resident microbiota contains primary degraders, cross-feeding partners, or taxa capable of competing for fermentation intermediates.

Several interpretive cautions are necessary. First, the term “enterotype” in this study refers to a pragmatic, genus-predominance grouping and should be distinguished from classical data-driven enterotypes such as *Bacteroides* spp., *Prevotella* spp., and *Ruminococcus* spp. enriched clusters [20]. This distinction should be made explicit because the four selected genera are health-relevant functional markers rather than universally validated enterotype anchors. Second, because only three representative donors from each group were included in the fermentation experiment, the findings should be interpreted as mechanistic and exploratory rather than fully generalizable to the broader population. Third, batch fermentation for 48 h is useful for screening substrate preference but does not reproduce the continuous substrate supply, absorption, pH control, host mucus, immune signaling, and spatial structure of the human colon. Finally, higher SCFA production and enrichment of beneficial taxa suggest prebiotic potential, but they do not by themselves prove a host health benefit under the ISAPP definition [10].

Overall, our data identify SAOS-1 as a promising AOS candidate with broad in vitro fermentability and enterotype-dependent microbiota-modulating capacity. These findings support the potential of SAOS-1 for precision prebiotic development, although animal and human intervention studies are needed to confirm its host health benefits. Future work should combine larger donor cohorts, shotgun metagenomics or metatranscriptomics, metabolomics, defined co-culture systems, and animal or human intervention studies to test whether SAOS-1 can reproducibly enhance SCFA production, strengthen gut barrier function, and reduce intestinal inflammation in vivo.

## 4. Materials and Methods

### 4.1. Chemicals and Reagents

Six types of AOS were obtained from the Marine Biomedical Research Institute of Qingdao (Qingdao, China), including two saturated alginate oligosaccharide fractions (SAOS-1 and SAOS-2), one OAOS, one UAOS, one SMOS, and one SGOS. Their structural features, including number-average molecular weight and average degree of polymerization, are summarized in Table 1 and Figure S2. Briefly, SAOS-1 and SAOS-2 were prepared from sodium alginate by enzymatic hydrolysis using a recombinant alginate lyase, followed by ultrafiltration and nanofiltration-based fractionation. SAOS-1 represented the higher-molecular-weight fraction, whereas SAOS-2 represented the lower-molecular-weight fraction. UAOS, OAOS, SMOS, and SGOS differed in saturation state, oxidation state, terminal chemistry, and M/G composition, as described in the Introduction and Table 1. These structural parameters were used to define the six AOS preparations for comparative fermentation analysis.

### 4.2. Fecal Sample Collection and Enterotype Classification

Fecal samples were collected from 32 healthy volunteers residing in Qingdao, China. All participants had no history of gastrointestinal disease or antibiotic treatment in the three months prior to sample collection. The study was approved by the Ethics Committee of Marine Biomedical Research Institute of Qingdao, Ocean University of China (Approval No. E-MBGY-2025-10-1), and all participants provided written informed consent.

Fresh fecal samples were collected anaerobically into sterile tubes. Total microbial DNA was extracted using the QIAamp DNA Stool Mini Kit (Qiagen, Hilden, Germany) according to the manufacturer’s protocol. The V3–V4 hypervariable region of the 16S rRNA gene was amplified using universal primers 338F (5′-ACTCCTACGGGAGGCAGCA-3′) and 806R (5′-GGACTACHVGGGTWTCTAAT-3′). Amplicon sequencing was performed on an Illumina MiSeq PE300 platform (San Diego, CA, USA) by Shanghai Majorbio Bio-pharm Biotechnology Co., Ltd. (Shanghai, China).

Bioinformatic analysis was carried out using the Majorbio Cloud platform (https://cloud.majorbio.com, accessed on 11 December 2025). Operational taxonomic units (OTUs) were clustered at 97% sequence similarity. Enterotypes were defined based on the predominant genus among four candidate genera: *Bifidobacterium* spp., *Bacteroides* spp., *Faecalibacterium* spp., and *Blautia* spp. Three representative fecal samples per enterotype (*n* = 3) were selected for subsequent in vitro fermentation experiments.

### 4.3. In Vitro Fermentation

In vitro batch fermentations were performed under strict anaerobic conditions (80% N_2_, 10% H_2_, and 10% CO_2_) at 37 °C using an AW500SG anaerobic chamber (Electrotek Ltd., Shipley, UK). A modified VI growth medium was prepared as previously described, containing (per liter): tryptone 3.0 g, peptone 3.0 g, yeast extract 4.5 g, bile salts No. 3 0.4 g, L-cysteine hydrochloride 0.8 g, hemin 0.05 g, NaCl 4.5 g, KCl 2.5 g, MgCl_2_·6H_2_O 0.45 g, CaCl_2_·6H_2_O 0.2 g, KH_2_PO_4_ 0.4 g, Tween 80 1 mL, and trace elements 2 mL. The six AOS were added to the medium at a final concentration of 8 g/L. A carbon-free blank control was also included. The medium was adjusted to pH 6.5 before autoclaving.

Fresh fecal samples were homogenized in sterile 0.1 M phosphate-buffered saline (PBS, pH 7.4) to prepare 20% (*w*/*v*) slurries, and large particles were removed using a 0.4 mm sieve. For each fermentation, 1 mL of fecal suspension was inoculated into 9 mL of VI medium containing the respective oligosaccharide. The cultures were sealed in Hungate tubes and incubated at 37 °C for 48 h in the anaerobic chamber. For each substrate and donor, fermentations were performed in three technical replicates. Three donors were included for each genus-predominance group and were treated as biological replicates. Representative donors were chosen based on the relative abundance of the dominant genus, overall community structure, and availability of sufficient fresh fecal material. This design was intended as a substrate-screening strategy rather than a population-level validation study.

### 4.4. High-Throughput Sequencing and Bioinformatic Analysis

After 48 h of fermentation, 5 mL of culture was centrifuged at 10,000× *g* for 10 min to collect bacterial pellets. Metagenomic DNA was extracted using the QIAamp DNA Stool Mini Kit. The V3–V4 region of the 16S rRNA gene was amplified and sequenced as described in Section 4.2.

Bioinformatic analysis was performed using the Majorbio Cloud platform. α-diversity indices (Chao1, ACE, Shannon, and Observed species) were calculated. β-diversity of the initial fecal microbiota was assessed by PCoA based on Bray–Curtis distance matrices calculated from OTU profiles and was used to visualize the separation of the four genus-predominance groups. PCA was additionally performed based on microbial community profiles to visualize overall community shifts before and after SAOS-1 fermentation. Linear discriminant analysis effect size (LEfSe) was applied to identify differentially abundant taxa among enterotype groups, with a logarithmic Linear discriminant analysis (LDA) score threshold of 2.0.

### 4.5. Total Carbohydrate Analysis and TLC

Total carbohydrate remaining in the fermentation medium was quantified using the phenol–sulfuric acid method. Briefly, fermentation samples were centrifuged at 10,000× *g* for 10 min to remove bacteria and insoluble particles. An aliquot (100 μL) of the supernatant was mixed with 100 μL of distilled water, followed by the addition of 200 μL of 6% (*w*/*v*) phenol and 1.5 mL of concentrated sulfuric acid. After incubation at 100 °C for 10 min, the absorbance was measured at 490 nm. The percentage of carbohydrate consumed after 48 h of fermentation was calculated relative to the 0 h control.

For TLC analysis, 0.2 μL of supernatant was loaded onto a silica gel-60 TLC aluminum plate (Merck, Darmstadt, Germany). The plate was developed in a solvent system of formic acid/n-butanol/water (6:4:1, *v*/*v*/*v*) for a distance of 10 cm. After drying, the plate was immersed in aniline–diphenylamine phosphate reagent and heated at 120 °C for 3 min to visualize carbohydrate spots.

### 4.6. SCFA Analysis

SCFAs in the fermentation medium were analyzed by high-performance liquid chromatography (HPLC). Fermentation samples were centrifuged at 14,000× *g* for 15 min, and the supernatant was filtered through a 0.22 μm membrane. Separation was achieved on an Aminex HPX-87H ion-exclusion column (300 × 7.8 mm, Bio-Rad, Hercules, CA, USA) using 5 mM H_2_SO_4_ as the mobile phase at a flow rate of 0.6 mL/min. The column temperature was maintained at 50 °C, and the eluate was monitored at 210 nm using an Agilent 1260 HPLC system (Agilent Technologies, Santa Clara, CA, USA). Acetate, propionate, and butyrate were quantified by comparison with standard curves of authentic standards.

### 4.7. Statistical Analysis

All data are presented as mean ± standard error of the mean (SEM). Statistical evaluations were performed using GraphPad Prism 9.0 (GraphPad Software, San Diego, CA, USA). One-way analysis of variance (ANOVA) followed by Tukey’s post hoc test was applied for multiple comparisons. Technical replicates were averaged before statistical analysis, and donor-level biological replicates were used as the independent statistical units. A *p*-value < 0.05 was considered statistically significant (* *p* < 0.05, ** *p* < 0.01, *** *p* < 0.001, and **** *p* < 0.0001).

## Figures and Tables

**Table 1:** Structural features of the six AOS preparations used in this study.

AOS Preparation	Structural Type	Mn	Average DP	Notes
SAOS-1	Saturated alginate oligosaccharide	810	4.1	Relatively higher-molecular-weight saturated AOS fraction
SAOS-2	Saturated alginate oligosaccharide	360	1.8	Relatively lower-molecular-weight saturated AOS fraction
UAOS	Unsaturated alginate oligosaccharide	640	3.2	Unsaturated AOS preparation
OAOS	Oxidized alginate oligosaccharide	870	4.4	Oxidized AOS preparation
SMOS	Saturated mannuronate oligosaccharide	980	4.9	Mannuronate-type saturated oligosaccharide
SGOS	Saturated guluronate oligosaccharide	700	3.5	Guluronate-type saturated oligosaccharide

Note: DP, degree of polymerization; Mn, number-average molecular weight; AOS, alginate oligosaccharides.

## Data Availability

The data presented in this study are available on request from the corresponding author.

## Supplementary Materials

The following supporting information can be downloaded at: https://www.mdpi.com/article/10.3390/md24070239/s1, Figure S1: Bacterial composition of each enterotype. Genus-level composition (A). Principal coordinate analysis (PCoA) (B); Figure S2. Schematic illustration of the theoretically chemical structures of AOS.

## Abbreviations

The following abbreviations are used in this manuscript:

AOS	Alginate oligosaccharides
SAOS	Saturated alginate oligosaccharides
SCFAs	Short-chain fatty acids
OAOS	Oxidized alginate oligosaccharides
UAOS	Unsaturated alginate oligosaccharides
SMOS	Saturated mannuronate oligosaccharides
SGOS	Saturated guluronate oligosaccharides
DP	Degree of polymerization
MW	Molecular weight
BF	Before fermentation
AF	After fermentation
OTUs	Operational taxonomic units
PCoA	Principal coordinate analysis
PCA	Principal component analysis
LEfSe	Linear discriminant analysis effect size
LDA	Linear discriminant analysis
TLC	Thin-layer chromatography
HPLC	High-performance liquid chromatography
PBS	Phosphate-buffered saline
ANOVA	Analysis of variance
SEM	Standard error of the mean
ISAPP	International Scientific Association for Probiotics and Prebiotics
