# Supplementary material for: Fermentation of Structurally Defined Alginate Oligosaccharides by the Human Gut Microbiota Enriched in Bifidobacterium, Bacteroides, Faecalibacterium, or Blautia
Source: Mar Drugs. 2026 Jul 7;24(7):239. doi: 10.3390/md24070239 (PMC13412613; doi:10.3390/md24070239)
Supplement: Supplementary file 1 [file marinedrugs-24-00239-s001.zip › marinedrugs-4378866-supplementary.pdf]

Supplementary Materials

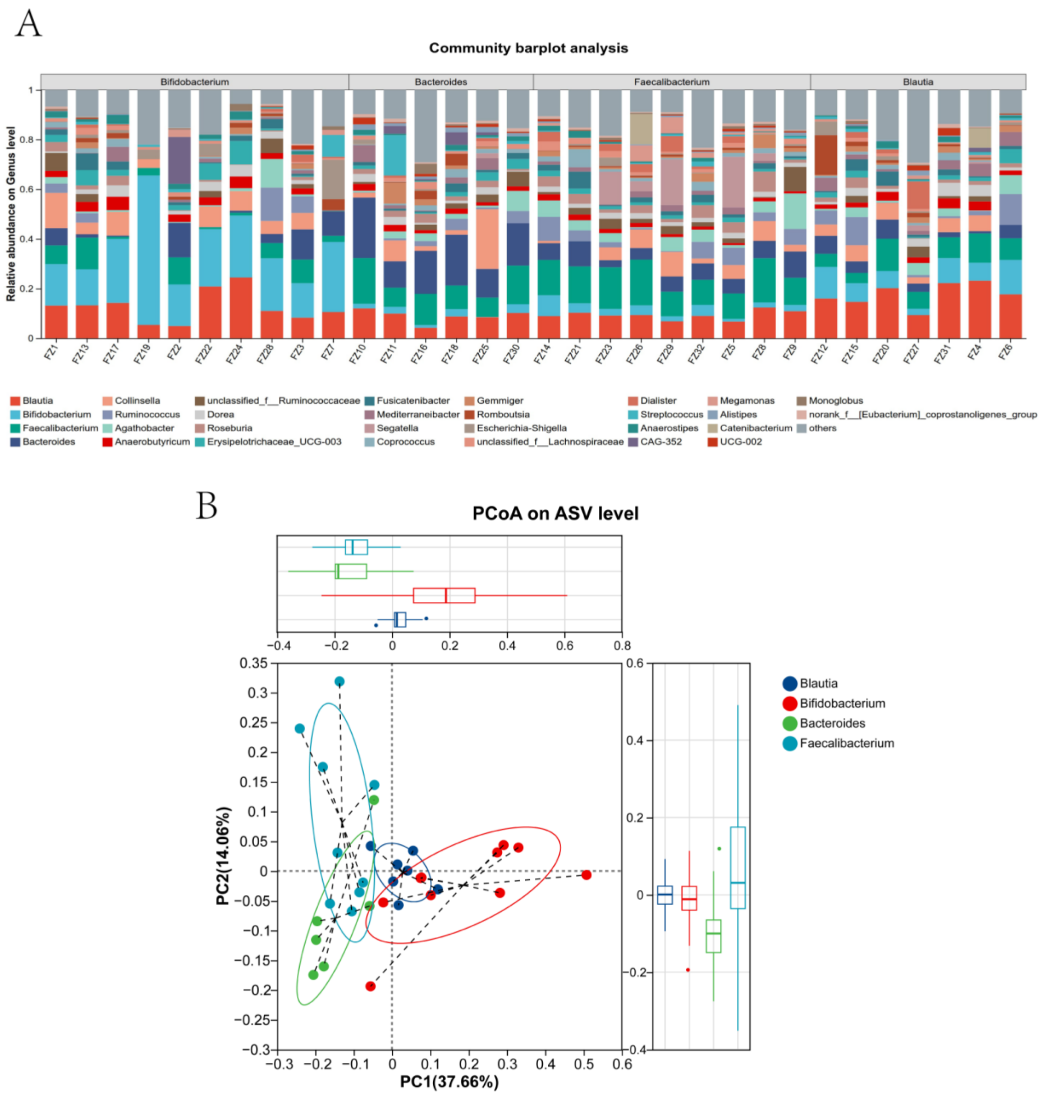

Figure S1. Bacterial composition of each enterotype. Genus-level composition (A). Principal coordinate analysis (PCoA) (B).

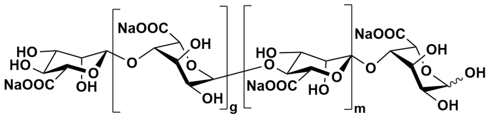

SAOS-1 , average DP=4.1
SAOS-2 , average DP=1.8

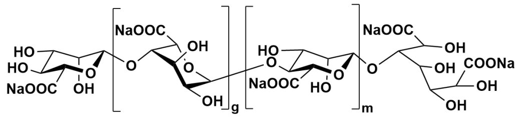

OAOS , average DP=4.4

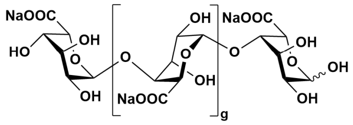

SGOS , average DP=3.5

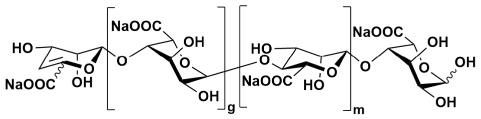

UAOS , average DP=3.2

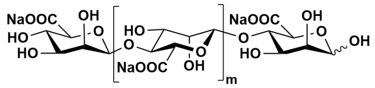

SMOS , average DP=4.9

Figure S2. Schematic illustration of the theoretically chemical structures of AOS.
